# Type II RAF inhibitor causes superior ERK pathway suppression compared to type I RAF inhibitor in cells expressing different BRAF mutant types recurrently found in lung cancer

**DOI:** 10.18632/oncotarget.24576

**Published:** 2018-02-27

**Authors:** Amir Noeparast, Philippe Giron, Sylvia De Brakeleer, Carolien Eggermont, Ulrike De Ridder, Erik Teugels, Jacques De Grève

**Affiliations:** ^1^ Laboratory of Molecular Oncology, Vrije Universiteit, Brussels, Belgium; ^2^ Department of Medical Oncology, Oncologisch Centrum, Universitair Ziekenhuis Brussel, Brussels, Belgium

**Keywords:** non-small cell lung cancer, Dabrafenib, Trametinib, AZ628, BRAF

## Abstract

A large fraction of somatic driver BRAF mutations in lung cancer are non-V600 and impaired-kinase. Non-V600 BRAF mutations predict sensitivity to combination of a type I RAF inhibitor, Dabrafenib, and a MEK inhibitor, Trametinib. Singly, Dabrafenib only weakly suppresses mutant BRAF-induced ERK signaling and can induce ERK paradoxical activation in CRAF-overexpressing cells. The present study compared the effects of Dabrafenib and a type II RAF inhibitor, AZ628, on ERK activity in HEK293T cells expressing several tumor-derived BRAF mutants, and in a non-V600 and impaired-kinase BRAF-mutant lung cancer cell line (H1666). Unlike Dabrafenib, AZ628 did not induce paradoxical ERK activation in CRAF-overexpressing cells and BRAF-mutant cells overexpressing CRAF were more responsive to AZ628 compared to Dabrafenib in terms of ERK inhibition. AZ628 inhibited ERK more effectively than Dabrafenib in both H1666 cells and HEK293T cells co-expressing several different BRAF-mutants with CRAF. Similarly, AZ628 plus Trametinib had better MEK-inhibitory and pro-apoptotic effects in H1666 cells than Dabrafenib plus Trametinib. Moreover, prolonged treatment of H1666 cells with AZ628 plus Trametinib produced greater inhibition of cell growth than Dabrafenib plus Trametinib. These results indicate that AZ628 has greater potential than Dabrafenib, both as a single agent and combined with Trametinib, for the treatment of non-V600 BRAF mutant lung cancer.

## INTRODUCTION

BRAF mutations are found in approximately 6–8% of non-small cell lung cancers (NSCLCs) [1–3]. As opposed to melanoma, in which about 90% of BRAF mutations are located at amino acid position V600, approximately half of all NSCLC BRAF mutations are predicted to be non-V600 [1, 2]. Since the advent of less restricted diagnostic methods, such as next-generation sequencing (NGS), the proportion of non-V600 BRAF mutations identified in different cancer types, including NSCLC, has grown [3]. BRAF mutations are generally classified as high kinase or impaired-kinase based on their kinase activity in cell-free assays. However, impaired-kinase BRAFs can still activate the extracellular signal-regulated kinase (ERK) pathway in cells through allosteric activation of CRAF, a dimerization partner of BRAF [4–6].

While therapeutic targeting of RAF and MEK via small molecule inhibitors has been clinically approved for V600E/K-mutated BRAF melanoma, the clinical application of these inhibitors in BRAF mutant NSCLC is at its early stages and requires further preclinical and clinical investigations [7, 8]. We recently showed that non-V600 BRAF mutations, independent of kinase activity status, predict sensitivity to the combination of the clinically available RAF-inhibitor, Dabrafenib, and MEK-inhibitor, Trametinib [6]. Most clinically available RAF inhibitors, including Dabrafenib and Vemurafenib, are type I inhibitors. Type I RAF inhibitors are ATP competitive and stabilize RAF in its active “DFG-in” conformation while blocking its catalytic activity [9–12]. Despite this inhibitory capacity, type I inhibitors induce dimerization of drug bound (B)RAF with CRAF, leading to allosteric activation of CRAF and paradoxical ERK activation [10, 13–15]. In wild type (WT) RAF and non-malignant cells, paradoxical ERK activation may result in development of cutaneous squamous cell carcinomas or keratoacanthomas [8, 14]. In some mutant BRAF homo/heterodimers (with CRAF), upon type I RAF inhibitor binding to mutant BRAF and induction of conformational change in the other protomer, the drug-free protomer can lose its affinity for the inhibitor or become transactivated (CRAF). For instance, one recurrent resistance mechanism in malignant V600E-mutated BRAF cells is restoration of homodimer signaling through amplification of the mutant allele (loss of affinity of drug-free protomer) or via heterodimer signaling through CRAF (CRAF transactivation in the presence of RAS) [15–17]. Some mutant forms of BRAF, such as high-kinase G469A BRAF, initially rely on homodimerization for oncogenic signaling and are poorly responsive to type I RAF inhibitor monotherapy (loss of affinity of drug-free protomer) [17].

Type II RAF inhibitors stabilize RAF in its “inactive” DFG-out conformation [18]. Although type II inhibitors can also induce RAF dimerization (CRAF homo and heterodimers with BRAF), they bind concomitantly to both RAF dimer partners and catalytically inhibit both protomers [19, 20].

We previously reported that non-V600E BRAF mutations, including impaired-kinase BRAFs, predict sensitivity to the combination of the type I RAF inhibitor Dabrafenib (2.5 μM), and MEK inhibitor Trametinib (25 nM) [6]. These concentrations are clinically relevant and achievable in patients given approved doses of Dabrafenib and Trametinib [21–22]. However, declining plasma concentration of RAF inhibitors over time and low tumor tissue concentrations may lead to unwarranted paradoxical ERK activation in patients harboring non-V600 BRAF mutants.

As different BRAF mutants can signal as innate or adaptive dimers, we hypothesized that type II RAF inhibition can suppress ERK signaling more efficiently than type I RAF inhibition. Therefore, we compared the ERK pathway inhibitory effects of the type II RAF inhibitor AZ628, with that of the type I RAF-inhibitor Dabrafenib, alone and in combination with Trametinib in mutant BRAF cells. We also compared the ERK inhibitory effects of both drugs in mutant BRAF HEK293T cells overexpressing CRAF. Finally, we compared the effects of AZ628 and Dabrafenib, both as single agents and in combination with Trametinib (MEK inhibitor), in a human NSCLC cell line (H1666) harboring a kinase-impaired BRAF mutation (G466V).

## RESULTS

### Effect of increasing Dabrafenib or AZ628 concentrations independently or in combination with Trametinib on BRAF-induced ERK pathway activity

We compared the effectiveness of Dabrafenib (type I inhibitor) and AZ628 (type II inhibitor) in HEK293T cells co-transfected with WT, D594N (impaired-kinase), or V600E (high-kinase) BRAF, and with CRAF. We performed titration experiments to evaluate potential paradoxical ERK activation associated with decreased inhibitor concentrations. We also compared the effects of these drugs in the presence of the MEK-inhibitor, Trametinib.

**Figure 1:**
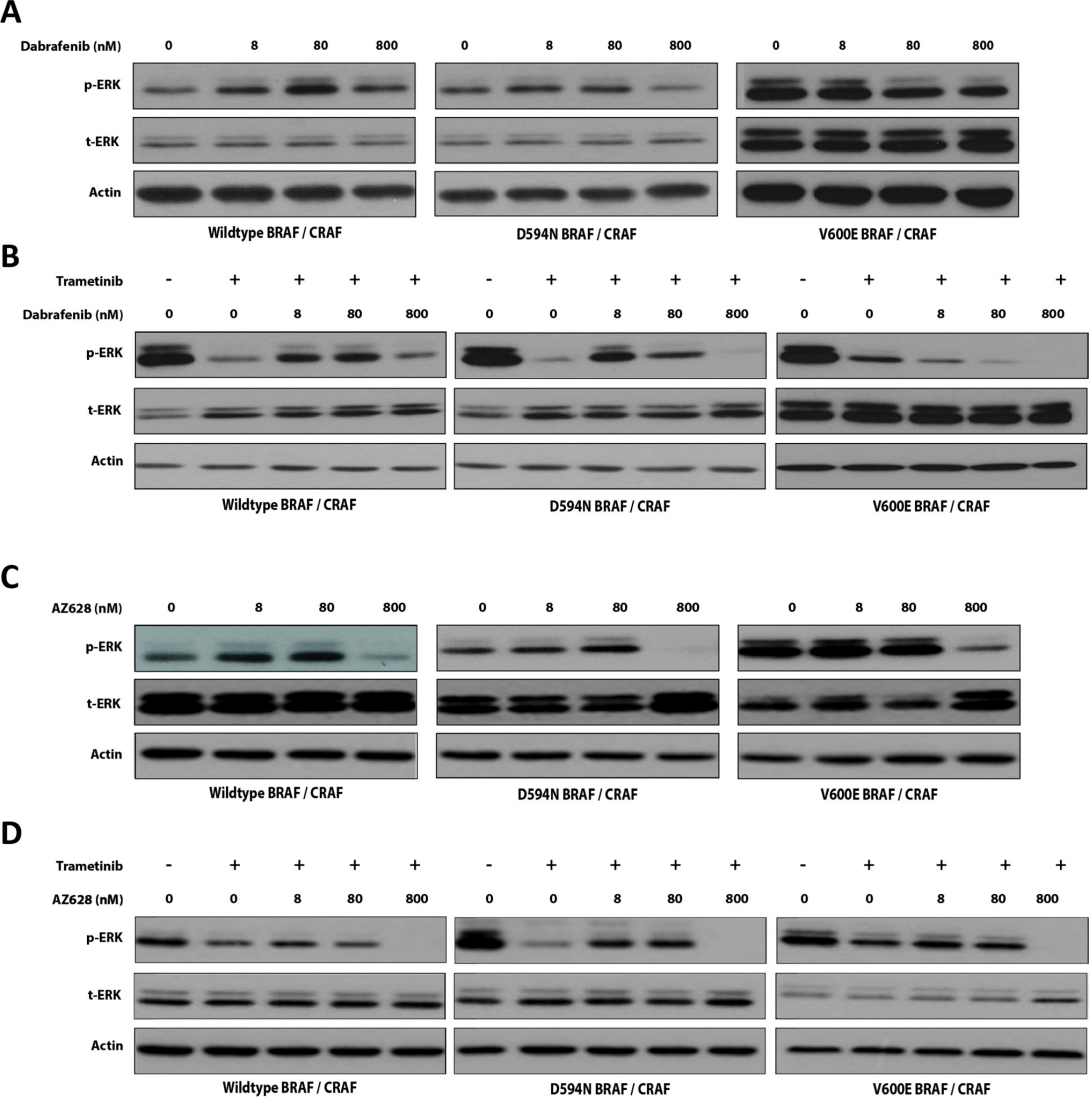
Effects of low-dose RAF inhibitors alone or in combination with Trametinib on BRAF-induced ERK pathway activity Effect of low-dose Dabrafenib (**A**) or AZ628 (**C**) on RAF-induced ERK signaling. BRAF expression vectors were co-transfected with CRAF into HEK293T cells. After 48 h, cells were incubated for 2 h with vehicle (DMSO) or incremental doses of Dabrafenib or AZ628. Whole cell lysates were subjected to Western blot analysis for ERK. Effect of Trametinib (25 nM) plus three incremented doses of Dabrafenib (**B**) or AZ628 (**D**) on BRAF-induced ERK activity. BRAF expression vectors were co-transfected with CRAF into HEK293T cells and treated with indicated inhibitors as in (A) and (C) Whole cell lysates were subjected to Western blot analysis for ERK.

In HEK293T cells co-expressing WT BRAF and CRAF, Dabrafenib increased ERK phosphorylation (p-ERK) at all tested concentrations (8, 80, and 800 nM) (Figure 1A). In contrast, AZ628 increased p-ERK only at the lowest concentrations (8 and 80 nM) and inhibited ERK at 800 nM (Figure 1C). Both drugs showed the strongest paradoxical ERK activation at 80 nM, although this effect was greater with Dabrafenib than AZ628. In D594N BRAF/CRAF co-expressing HEK293T cells, both Dabrafenib and AZ628 treatment slightly increased p-ERK levels at 8 and 80 nM, and downregulated p-ERK at 800 nM (Figure 1A and 1C). The strongest ERK inhibition was again observed upon AZ628 treatment. In V600E BRAF/CRAF co-expressing HEK293T cells exposed to Dabrafenib (8, 80 and 800 nM), ERK inhibition increased proportionally with increasing Dabrafenib concentration (Figure 1A). In these cells, AZ628 only altered p-ERK levels at 800 nM (Figure 1C).

We previously showed that BRAF mutations, irrespective of mutation type, predict sensitivity to the combination of Dabrafenib and Trametinib at conventional doses (25 nM for Trametinib and 2500 nM for Dabrafenib) [6]. To evaluate whether the observed paradoxical ERK activation by lower doses of single agents would influence ERK suppression by the combination therapies, we evaluated the effects of low-dose Dabrafenib or AZ628 (8, 80, and 800 nM) in combination with Trametinib (25 nM) in HEK293T cells co-transfected with WT, D549N-, or V600E-mutated BRAF together with CRAF.

In WT BRAF/CRAF co-expressing HEK293T cells, Trametinib therapy alone strongly inhibited ERK (Figure 1B and 1D). When combined with low-dose Dabrafenib (8 or 80 nM), Trametinib-induced ERK inhibition was antagonized. Consistent with Dabrafenib monotherapy results, this effect was maximal at 80 nM Dabrafenib. At 800 nM, Dabrafenib did not alter the effect of Trametinib on ERK (Figure 1B). In cells treated with AZ628 and Trametinib, the antagonistic effect was less pronounced than in Dabrafenib and Trametinib treated cells (Figure 1D). Additionally, ERK inhibition was most strongly enhanced at 800 nM AZ628 in combination with Trametinib.

In D594N BRAF/CRAF co-transfected cells, Dabrafenib at 8 and 80 nM antagonized Trametinib-induced ERK inhibition (Figure 1B), and this effect was greatest at 8 nM. In contrast, Trametinib with 800 nM Dabrafenib suppressed ERK activity to lower levels than Trametinib alone (Figure 1B). Despite strong Dabrafenib antagonization of Trametinib, co-treatment always resulted in ERK inhibition compared to the vehicle group. Similar to Dabrafenib, AZ628 antagonized Trametinib in these cells at 8 and 80 nM, but not at 800 nM (Figure 1D).

In V600E BRAF/CRAF co-transfected cells, addition of Dabrafenib to Trametinib enhanced ERK inhibition at all three doses, proportional to increasing Dabrafenib concentrations. ERK inhibition was the same in cells treated with Trametinib alone or AZ628 (8 and 80 nM) plus Trametinib. However, ERK suppression was enhanced in cells treated with Trametinib plus 800 nM AZ628 (Figure 1D).

### AZ628 does not induce paradoxical ERK activation in WT RAF-expressing HEK293T cells

**Figure 2:**
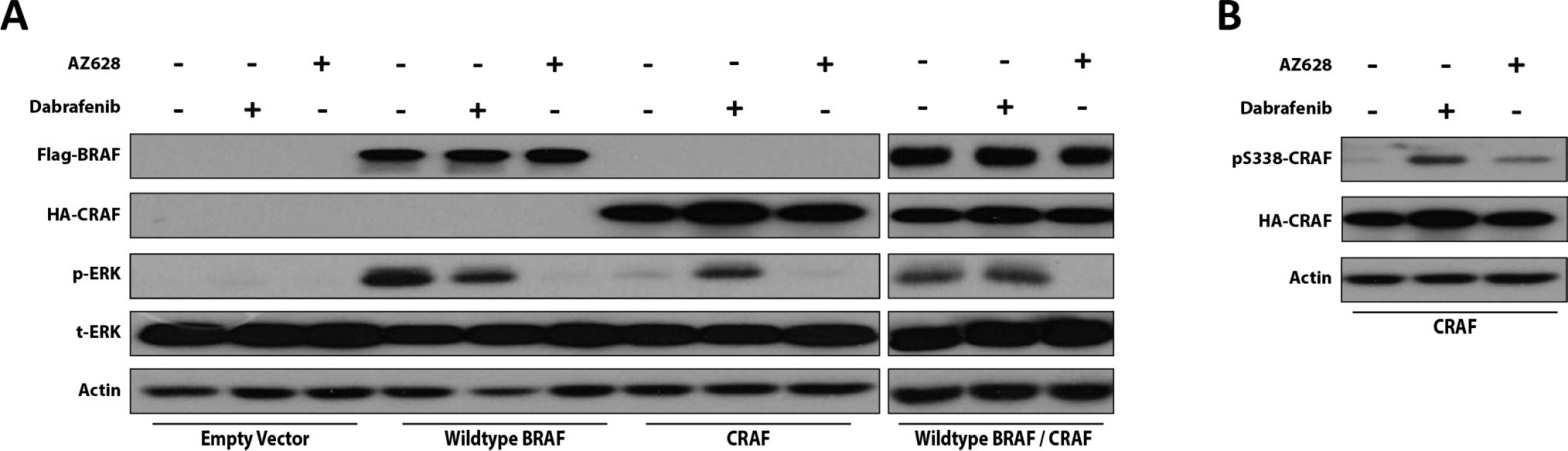
Dabrafenib and AZ628 effects on ERK activity in WT RAF-expressing HEK293T cells HEK293T cells were transiently transfected or co-transfected with WT BRAF, WT CRAF, or WT BRAF/WT CRAF expression vectors. 48 h post-transfection, cells were treated for 2 h with DMSO, Dabrafenib (2.5 μM), or AZ628 (2.5 μM), then lysed and subjected to Western blot analysis for the indicated proteins (**A**) Effects of Dabrafenib and AZ628 on CRAF S338 phosphorylation in CRAF-expressing HEK293T cells (**B**).

To further investigate the capacity of Dabrafenib and AZ628 to induce paradoxical ERK activation, we compared ERK activation between WT BRAF-expressing, CRAF-expressing, and WT BRAF/CRAF co-expressing HEK293T cells following 2 h AZ628 treatment. Both drugs were evaluated at 2.5 μM. Notably, the maximal plasma concentration of Dabrafenib at approved clinical doses is 2.5–4 μM [21]. In BRAF-expressing cells, both Dabrafenib and AZ628 treatments inhibited ERK, and this effect was more pronounced with AZ628 treatment (Figure 2A). In cells transfected with CRAF alone, Dabrafenib, but not AZ628, induced paradoxical ERK activation. Dabrafenib did not inhibit ERK in BRAF/CRAF co-expressing cells, while AZ628 treatment resulted in strong ERK inhibition. These results indicated that, unlike Dabrafenib, AZ628 does not induce ERK paradoxical activation in CRAF overexpressing cells.

CRAF S338 phosphorylation has been associated with conformational activation of CRAF [14, 23, 24]. To compare the capacities of Dabrafenib and AZ628 to conformationally transactivate RAF protomers, we evaluated CRAF S338 phosphorylation status following Dabrafenib and AZ628 treatment in CRAF-expressing HEK293T cells (Figure 2B). As expected, Dabrafenib treatment increased CRAF S338 phosphorylation, while AZ628 treatment only slightly elevated CRAF S338 phosphorylation (Figure 2B).

### Increased CRAF expression desensitizes BRAF-mutant HEK293T cells to Dabrafenib, but not to AZ628

**Figure 3:**
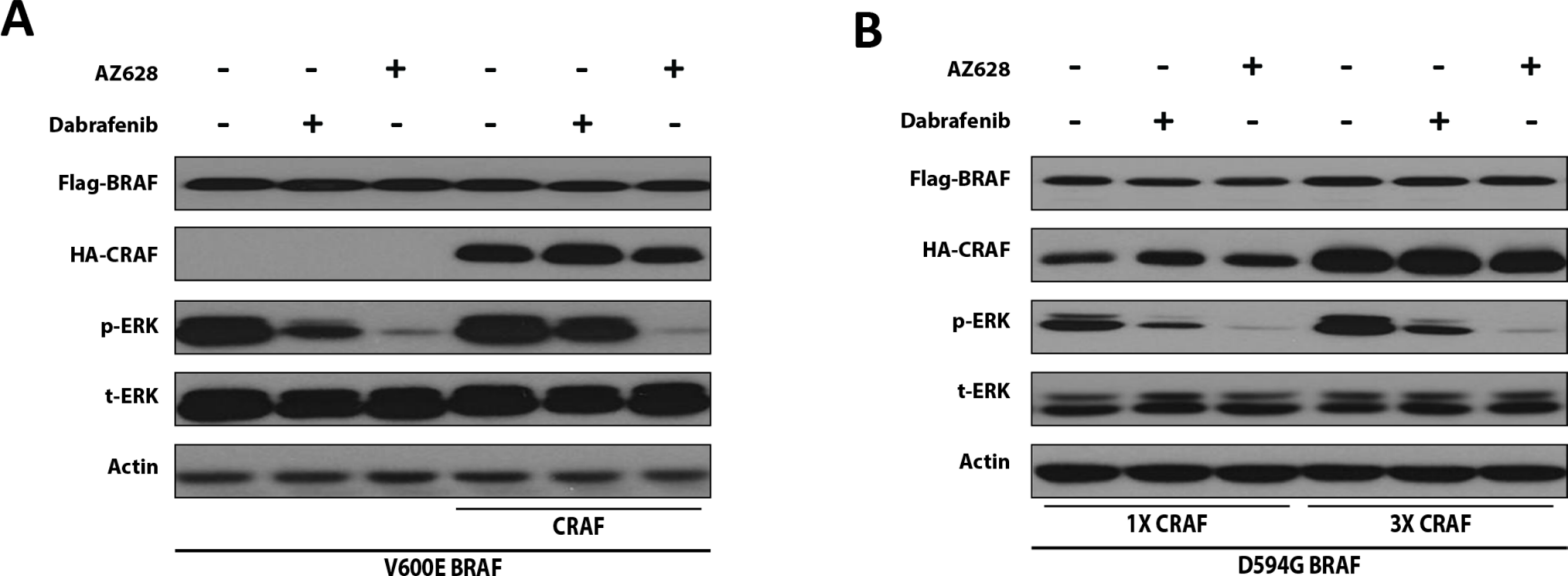
CRAF overexpression reduces the ERK-inhibitory effect of Dabrafenib, but not AZ628 in mutant BRAF-expressing HEK293T cells HEK293T cells were transiently transfected with V600E BRAF (0.2 μg) alone or with CRAF (0.6 μg) (**A**) or with a constant amount of impaired-kinase D594G BRAF expression plasmid and increasing amounts of CRAF expression plasmid (**B**) 48 h post-transfection, cells were treated for 2 h with DMSO, Dabrafenib (2.5 μM), or AZ628 (2.5 μM), then lysed and subjected to Western blot analysis.

To further examine the impact of CRAF expression on the ERK-inhibitory effects of Dabrafenib and AZ628 in the context of a high-kinase BRAF mutation, we co-transfected HEK293T cells with V600E BRAF (high-kinase BRAF) and CRAF (or the corresponding empty vector). CRAF expression reduced Dabrafenib-mediated ERK inhibition, but barely altered AZ628-mediated ERK inhibition (Figure 3A).

To evaluate the impact of CRAF expression on Dabrafenib- or AZ628-mediated ERK inhibition in the context of impaired-kinase BRAF, we co-transfected HEK293T cells with D549G BRAF (impaired-kinase BRAF) together with low or high levels of CRAF plasmid. Cells were treated with Dabrafenib, AZ628, or mock. Increased CRAF expression reduced Dabrafenib-, but not AZ628-mediated ERK inhibition in D549G BRAF cells (Figure 3B).

### AZ628 inhibits ERK more effectively than Dabrafenib in HEK293T cells co-expressing CRAF and mutant BRAF

**Figure 4:**
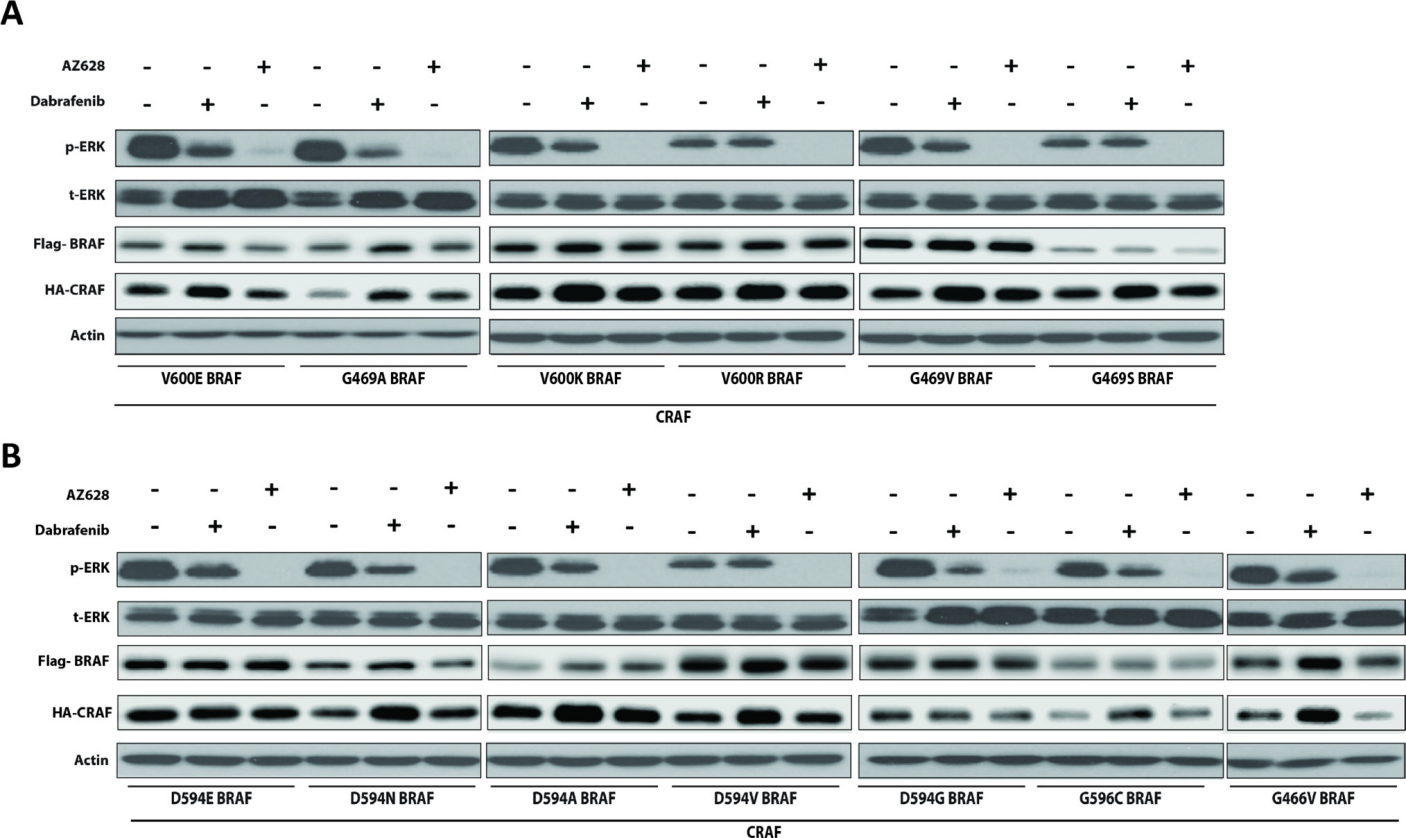
Dabrafenib and AZ628 effects on ERK activity in HEK293T cells co-expressing various mutant BRAFs together with CRAF HEK293T cells were transiently co-transfected with various recombinant BRAF expression vectors together with CRAF. 48 h post-transfection, cells were treated for 2 h with DMSO, Dabrafenib (2.5 μM), or AZ628 (2.5 μM), then lysed and subjected to Western blot analysis. Upper panel (**A**) includes high-kinase BRAF mutants. Lower panel (**B**) includes impaired-kinase BRAF mutants. Inhibitory effects observed with AZ628 were always stronger than with Dabrafenib. Slight CRAF upregulation was observed upon Dabrafenib treatment, which may be associated with Dabrafenib binding to CRAF [52].

To determine whether our findings with representative BRAF mutants could be extrapolated to other previously described BRAF mutants, we co-transfected HEK293T cells with 13 different patient-derived impaired and high-kinase BRAF mutants (see Table 1 in [6]) together with CRAF, and examined ERK activation status 2 h post-treatment with conventional Dabrafenib (2.5 μM) or AZ628 (2.5 μM) doses (Figure 4A–4B). Both inhibitors decreased p-ERK1/2 levels in HEK293T cells co-expressing CRAF with BRAF mutants conferring elevated or impaired-kinase activity. AZ628 treatment more strongly inhibited ERK than Dabrafenib treatment. Dabrafenib treatment did not inhibit ERK in D594V BRAF cells (Figure 4B) [6]. These results suggest that AZ628 inhibits ERK more effectively than Dabrafenib in BRAF mutant cells, independent of mutation type.

### Dabrafenib versus AZ628 when combined with Trametinib in an impaired-kinase BRAF mutant NSCLC cell line (H1666)

Non-V600 BRAF mutations with impaired-kinase activity are frequently found in NSCLC. Little is known about drug responses and possible resistance mechanisms in cells with these mutations. Therefore, we further compared AZ628 and Dabrafenib in a kinase impaired non-V600 BRAF NSCLC cell line. Unfortunately, only two NSCLC cell lines harboring a kinase-impaired BRAF mutation have been documented and both harbor the G466V mutation (H1666 and CAL-12). CRAF knockdown arrests growth by more than 50% in H1666 cells (G466V-BRAF, heterozygous), versus 20% in CAL-12 cells [25]. Thus, we chose H1666 cells to compare the effects of AZ628 and Dabrafenib as single agents and in combination with Trametinib on the ERK pathway, apoptosis induction, and cell viability.

**Figure 5:**
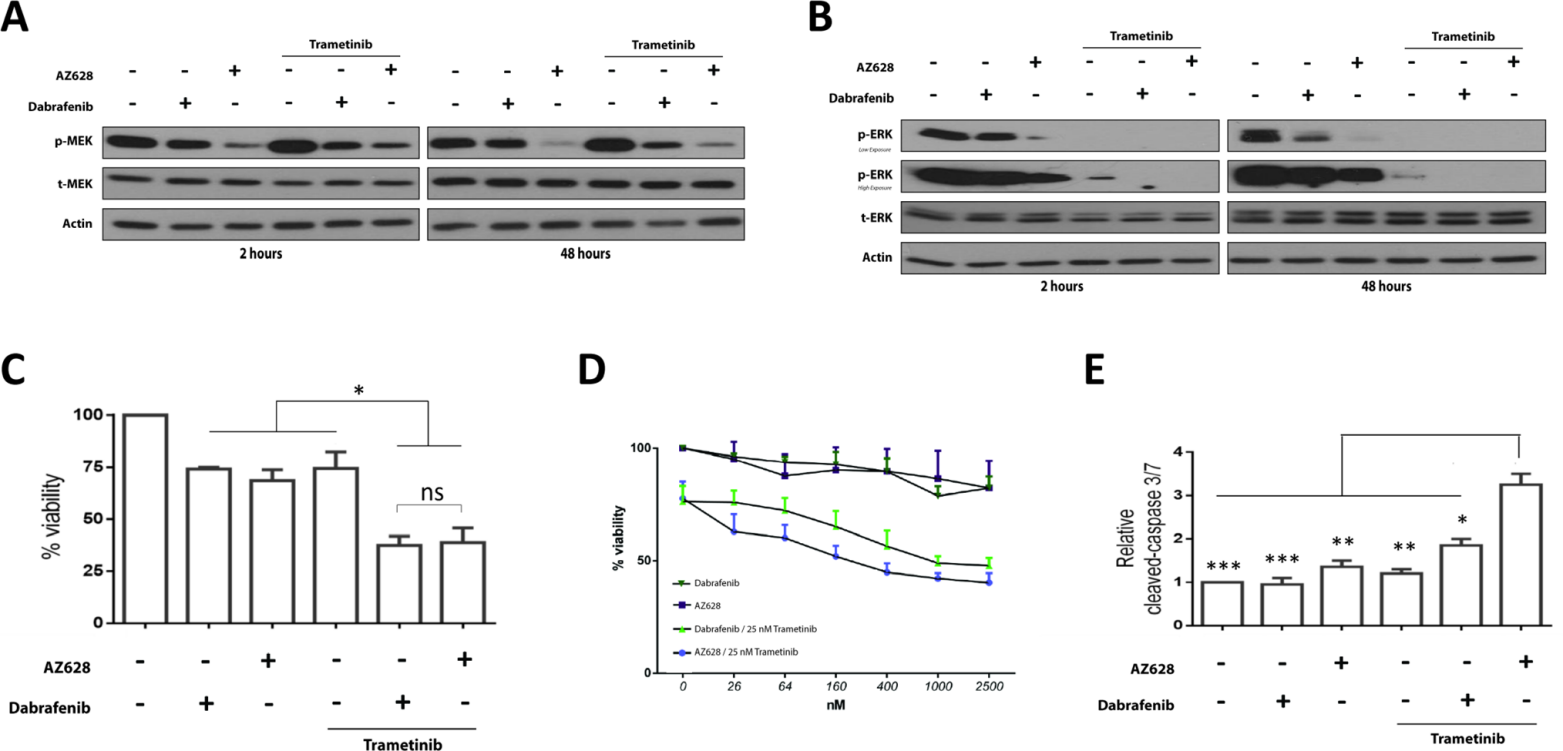
Effects of Dabrafenib, AZ628, and Trametinib alone or in combination on H1666 cells H1666 Cells were incubated for 48 h with Dabrafenib (2.5 μM), AZ628 (2.5 μM), or Trametinib (25 nM) alone or in combination (Dabrafenib or AZ628 plus Trametinib). Whole cell lysates were subjected to Western blot analysis (**A** and **B**). H1666 cells were incubated for three days with Dabrafenib (2.5 μM), AZ628 (2.5 μM), or Trametinib (25 nM) alone or in combination (Dabrafenib or AZ628 plus Trametinib). Viability was measured, and relative viability was determined via normalization to the vehicle group (**C**). Means ± SEM are from two independent experiments, each performed in six replicates. All three inhibitors showed comparable effects as single agents, and combined RAF/MEK inhibitor treatments were more efficient. H1666 cells were incubated for three days with incremental doses of Dabrafenib and AZ628 and a constant Trametinib dose (25 nM). Viability was measured, and relative viability was determined via normalization to the vehicle group (**D**). Means ± SEM are from three independent experiments, each performed in four replicates. Cells were incubated for three days as in (D) and Caspase-3/7 activity was measured and normalized to the number of viable cells (**E**). Single agent treatments had no pro-apoptotic effects. AZ628 plus Trametinib showed a stronger pro-apoptotic effect than Dabrafenib plus Trametinib. Values are displayed as fold increase compared to the vehicle group. Means ± SEM are from two independent experiments, each performed in six replicates. ^*^p ≤ 0.05, ^**^p ≤ 0.01, ^***^p ≤ 0.001.

We treated H1666 cells with 2.5 μM Dabrafenib or AZ628 for 2 and 48 h (Figure 5A–5B). At both time points, AZ628 induced stronger MEK and ERK inhibition than Dabrafenib. Trametinib treatment alone increased MEK phosphorylation and decreased downstream p-ERK levels. AZ628 or Dabrafenib plus Trametinib enhanced MEK and ERK inhibition compared to single treatments. Both combination treatments suppressed ERK below detection limits.

### Dabrafenib and AZ628 reduce H1666 cell proliferation, and Trametinib enhances this effect

We compared the effects of Dabrafenib and AZ628 in H1666 cells at conventional doses (Figure 5C) and at concentrations (Figure 5D) ranging from 26 nM–2.5 μM, alone or in combination with Trametinib (25nM). The lower concentrations were selected to verify whether paradoxical ERK activation, as observed in HEK293T cells, could influence cell viability. Viability was measured after 72 h incubation (Figure 5C–5D). Dabrafenib or AZ628 alone had comparable effects on cell viability. At 2.5 μM Dabrafenib or AZ628 we observed 74 ± 0.86% and 68 ± 5.2% viable cells (% viable cells ± SEM), respectively, compared to controls (Figure 5C). In combination with Trametinib, AZ628 and Dabrafenib (Figure 5C) showed comparable cell growth inhibitory effects ( 40.3 ± 4.2% and 47.8 ± 3.4% viable cells, respectively, 72h after treatment). At lower doses, both AZ628 and Dabrafenib as single agents (Figure 5D) produced similar, limited declines in viability. AZ628 plus Trametinib resulted in a stronger growth inhibitory effect than Dabrafenib plus Trametinib, although this result was not significant (Figure 5D).

### AZ628 plus Trametinib has superior pro-apoptotic effects in H1666 cells compared to Dabrafenib plus Trametinib

To evaluate whether single or combined treatments trigger apoptosis, we measured caspase 3/7 activation after 72 h treatment. No single agent resulted in caspase 3/7 activation compared to controls (Figure 5E). In combination with Trametinib, both Dabrafenib and AZ628 increased caspase 3/7 activity compared to controls and single agents, and this effect was greatest after treatment with AZ628 plus Trametinib (Figure 5E).

### Prolonged treatment of H1666 cells with AZ628 plus Trametinib leads to greater growth inhibition than Dabrafenib plus Trametinib

**Figure 6:**
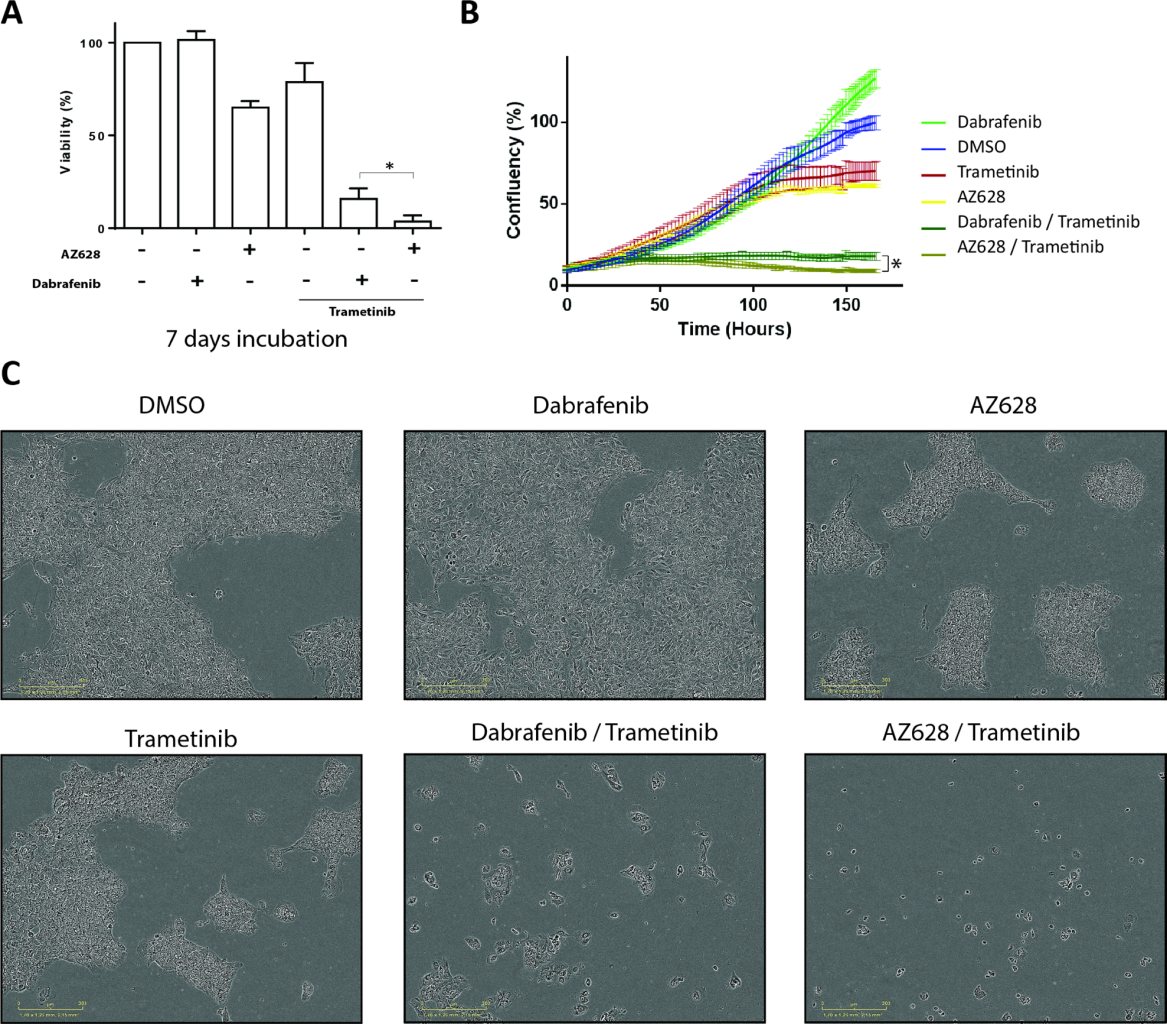
Prolonged treatment of H1666 cells with Dabrafenib, AZ628, and Trametinib alone or in combination H1666 cells were incubated for seven days with Dabrafenib (2.5 μM), AZ628 (2.5 μM), or Trametinib (25 nM) alone or in combination (Dabrafenib or AZ628 plus Trametinib). Viability was measured, and relative viability was determined via normalization to the vehicle group (**A**). Means ± SEM are from four independent experiments, each performed in four replicates. Alternatively, cells treated as described were incubated and monitored in an Incucyte device and confluency was determined at several time points (**B**). Images representative of different conditions in (B) were taken after seven days (**C**). ^*^p ≤ 0.05, ^**^p ≤ 0.01, ^***^p ≤ 0.001.

The superior pro-apoptotic effect of AZ628 (2.5 μM) plus Trametinib (25 nM) versus Dabrafenib (2.5 μM) plus Trametinib (25 nM) in H1666 cells after 72 h treatment was not associated with decreased cell viability (Figure 5C and 5E). We further evaluated the long-term effects of these drugs on cell growth at conventional doses. We measured cell confluency over one week using periodical phase contrast imaging via the Incucyte system, followed by an end-point analysis using the CellTiter-Glo Luminescent Cell Viability Assay. H1666 cell incubation with Dabrafenib alone for one week did not result in decreased cell viability, these cells reached even higher confluencies compared to DMSO controls. This increased confluency was associated with a less dense distribution of cells compared to controls and AZ628-treated cells (Figure 6A–6C and Supplementary Figure 1). In contrast to Dabrafenib and consistent with 72 h treatment results, one week of treatment with either AZ628 or Trametinib alone decreased H1666 cell confluency as well as viability (to 65% and 78.7%, respectively) compared to DMSO controls. Moreover, one-week treatment of H1666 cells with AZ628 plus Trametinib vs. Dabrafenib plus Trametinib decreased cell viability by 15.75% vs. 3.5% and confluency by 18% vs. 9%, respectively (Figure 6A–6C).

## DISCUSSION

This study compared the type I RAF inhibitor, Dabrafenib, and the type II RAF inhibitor, AZ628, as single agents and in combination with the MEK inhibitor, Trametinib, in both transfected HEK293T cells overexpressing several RAF derivatives and a BRAF mutant NSCLC derived cell line model. V600E mutant melanoma cells were previously shown to develop resistance to AZ628 treatment upon elevated CRAF expression [26]. However, our work is the first to compare type I and II RAF inhibitors in the presence of elevated CRAF in cells expressing different types of BRAF mutants, including impaired-kinase mutants. In the HEK293T cell model, we showed that BRAF mutations recurrently found in NSCLC, irrespective of mutation type, predict better sensitivity to AZ628 versus Dabrafenib. In H1666 cells, we found that the combination of AZ628 and Trametinib was superior to Dabrafenib and Trametinib (in terms of cell growth inhibition, pro-apoptotic marker induction and MEK inhibition) .

In the HEK293T model, CRAF transfectants exhibited lower ERK activity than both WT BRAF-expressing and WT BRAF/CRAF co-expressing cells, indicating that CRAF on itself is kinase inactive in this condition. However, upon Dabrafenib treatment, CRAF was activated and induced downstream signaling leading to paradoxical ERK activation. This was consistent with previous reports on type I RAF inhibitor-induced paradoxical ERK activation via induction of CRAF homodimerization and transactivation [6, 11, 27]. Moreover, RAF inhibitor-induced dimerization (type I) results in formation of asymmetric RAF dimers in which one of the protomers loses its affinity for the inhibitor and is also catalytically transactivated [11, 14, 17, 24]. Earlier studies suggest that both Dabrafenib and AZ628 induce RAF dimerization [28]. We observed AZ628-induced phosphorylation of CRAF at residue S338, indicating that CRAF conformational activation [14, 29–31] also occurs with AZ628. However, AZ628 induced less CRAF phosphorylation than Dabrafenib. In spite of CRAF S338 phosphorylation, we did not observe paradoxical ERK activation upon AZ628 treatment. These findings suggest that upon AZ628-induced CRAF conformational activation, both CRAF catalytic clefts maintain affinity for the inhibitor and both CRAF protomers are catalytically inhibited. Thus, single agent AZ628 treatment of WT RAF-expressing cells is less likely than Dabrafenib to induce paradoxical ERK activation-related adverse events. CRAF S338 phosphorylation has been suggested to be linked to ERK-independent resistance to RAF-inhibition [32]. Yet, contribution of CRAF S338 phosphorylation mediated by either types of RAF-inhibitors in ERK-independent resistance mechanisms to these inhibitors has to be further clarified [32–35].

Whittaker, *et al.* showed that CRAF expression generates resistance to type I RAF inhibitors in V600E BRAF mutant colorectal cancer [19]. Similarly, we found that CRAF overexpression in V600E BRAF HEK293T cells diminished Dabrafinib-mediated ERK inhibition, while AZ628 retained strong ERK-inhibitory effects.

In cell-free assays, type I RAF inhibitors equally inhibit both WT RAF isoforms and V600E BRAF [27, 36, 37]. Dabrafenib inhibits both V600E BRAF and CRAF kinase activity at IC50s lower than those of AZ628 [19, 27, 37, 38]. In the cellular context, however, type I RAF inhibitors do not exhibit comparable CRAF inhibitory effects. Apparent Km for ATP is much less for CRAF than for V600E BRAF [19]. At cellular (higher) ATP concentrations, the Dabrafinib IC50 for CRAF is strongly increased while that for V600E BRAF remains low. This explains why type I RAF inhibitors are not equipotent inhibitors of V600E and CRAF in the cellular context, and is consistent with our observation regarding the superior efficacy of AZ628 versus Dabrafenib in the presence of CRAF.

We observed that AZ628 was a much stronger ERK pathway inhibitor than Dabrafenib in HEK293T cells overexpressing V600E BRAF, even in the absence of CRAF. This contradicts the assumptions that in cells, Dabrafenib always can potently inhibit V600E BRAF in the absence of CRAF. Recent studies found that V600E BRAF overexpression (e.g. due to gene amplification) can restore V600E homodimer signaling under type I RAF-inhibitor treatment [16, 17], a condition mimicked by our mutant V600E BRAF-overexpressing HEK293T cells. Consequently, the better response observed with AZ628 compared to Dabrafenib was predictable since type II inhibitors, such as AZ628, have reduced potential to transactivate RAF dimers upon binding to one protomer (early adaptive insensitivity mechanism seen for type I inhibitors) and they do not reduce the affinity of unbound protomer for the inhibitor (homodimer signaling) [17, 19]. Moreover, AZ628 has a very slow off-rate and irreversibly inhibits RAF [24, 39]. AZ628 is thus superior to Dabrafenib with respect to adverse events related to ERK paradoxical activation, adaptive homodimer signaling, and early adaptive insensitivity.

Impaired-kinase BRAF mutants rely on dimerization and allosteric activation of CRAF for ERK pathway activation [4, 14]. We previously showed that Dabrafenib inhibits impaired-kinase BRAF-induced and CRAF-mediated ERK pathway activity [6]. Our current study found that AZ628 more potently suppresses impaired-kinase BRAF-induced ERK activation than Dabrafenib. Our results suggest that AZ628 has higher cellular affinity for catalytically active CRAF in the impaired-kinase BRAF-CRAF heterodimer complex than Dabrafenib, resulting in stronger ERK pathway inhibition.

We observed that CRAF overexpression diminished Dabrafenib-, but not AZ628-mediated ERK pathway inhibition in impaired-kinase BRAF transfected cells. This effect is likely explained by the increased fraction of CRAF protomeres not involved in heterodimerization with mutant BRAF molecules. This increased concentration of CRAF protomeres can then contribute to a larger pool of CRAF homodimers, which in the presence of type I inhibitor can lead to paradoxical ERK activation [13, 14]. AZ628 does not induce paradoxical ERK activation, suggesting that even in the presence of excessive amounts of CRAF, the overall inhibitory effect of AZ628 in impaired-kinase BRAF expressing cells remains unaffected.

A category of high-kinase BRAF mutants, including the recurrent G469A variant, function as constitutively active homodimers [17]. These homodimers also appear insensitive or poorly responsive to type I RAF inhibition [17]. However, we found that AZ628 was superior to Dabrafinib in inhibiting the ERK pathway in HEK293T cells expressing different high-kinase BRAF mutants together with CRAF. These findings suggest that tumors harboring high-kinase BRAF mutant forms that signal as homodimers will respond better to type II RAF-inhibitors, even in the presence of CRAF.

Our previous study showed that different lung-derived BRAF mutants (non-V600) predict sensitivity to the combination of Dabrafenib and Trametinib. Our present work assessed the potency of AZ628 versus Dabrafenib in combination with Trametinib as measured by cell viability, caspase 3/7 activation, and ERK pathway activation in a tumor derived cell line harboring an impaired kinase mutation (H1666 cell line) both short term (24–48h) and long term (1 week).

Surprisingly, the MEK inhibitor Trametinib alone upregulated p-MEK in H1666 cells and decreased downstream ERK phosphorylation. A similar phenomenon was previously described in A549, a KRAS-mutant NSCLC cell line (in which ERK activation is CRAF-mediated as opposed to BRAF-mediation in V600E mutant cells) [40–42]. Using a p-MEK antibody similar to ours (recognizing both MEK phosphorylated sites at S221 and S217) combined with mass spectrometry, Gilmartin *et al.* [40] could conclude that Trametinib inhibits MEK S217 phosphorylation and increases S221 phosphorylation. In another study, Lito, *et al.* [41] showed that in A549 cells most MEK inhibitors induce MEK-CRAF complex formation, resulting in further reactivation of the inhibited MEK by active CRAF and consequently insensitivity to MEK inhibitors. It was subsequently shown that CRAF inhibition is required for more efficient MEK inhibition in KRAS mutant cells (and not in V600E BRAF mutant cells) [41, 42]. We found that AZ628 plus Trametinib more strongly inhibited MEK than Dabrafenib plus Trametinib in impaired-kinase BRAF NSCLC cells. Because ERK activation is CRAF-mediated [6, 25] in H1666 cells and AZ628 inhibits CRAF more effectively than Dabrafenib, CRAF inhibition likely enhances Trametinib-mediated MEK inhibition.

H1666 cells were poorly responsive to single-agent Dabrafenib and developed resistance after prolonged (7 days) Dabrafenib monotherapy. Indeed, cells treated with Dabrafenib exhibited higher confluencies than even DMSO-treated controls, although viability assay results could not confirm this difference. Confluency differences between DMSO- and Dabrafenib-treated cells might thus be due to cell distribution alterations (resulting in higher covered areas/cell) rather than increased cell numbers, as supported by phase-contrast microscopy imaging. In KRAS mutant cells in which ERK pathway activation is CRAF-dependent, type I RAF inhibitors can induce ERK paradoxical activation and subsequently increased cell proliferation [9]. Our results predict that this may also occur upon long-term Dabrafenib monotherapy in H1666 cells.

After 72 h, both drug combinations produced a comparable percentage of viable H1666 cells, although AZ628/Trametinib treatment produced higher pro-apoptosis rates. Notably, at lower doses, AZ628/Trametinib appeared to inhibit cell growth more than Dabrafenib/Trametinib. Whittaker, *et al.* also found that AZ628 had high efficacy when combined with another MEK inhibitor in V600E BRAF colorectal cancer cell lines [19]. H1666 cells are responsive to Dabrafenib/Trametinib and therefore cannot serve as a Dabrafenib/Trametinib-resistant cell model. However, our results suggest that further CRAF upregulation would likely desensitize CRAF-dependent cells, such as H1666, to Dabrafenib/Trametinib, but not (or to lesser extent) to AZ628/Trametinib. Moreover, prolonged incubation of H1666 cells revealed that AZ628/Trametinib inhibited cell growth more effectively than Dabrafenib/Trametinib.

Toxicity related to ERK pathway inhibition in WT RAF cells is a potential concern for type II pan-RAF inhibitors. However, clinical studies with the FDA-approved inhibitor Sorafenib (a type II pan-RAF inhibitor but also multiple kinase inhibitor), have not revealed prohibitive toxicities related to type II RAF inhibition [43, 44]. Additionally, emerging type II RAF dimer inhibitors may have a wide therapeutic index [20, 28]. Both type I and II RAF inhibitors as single agents are prone to ERK pathway-related resistance mechanisms, and MEK inhibitors can fail in cells with CRAF-mediated ERK activation [9, 11, 13, 24, 26, 41, 42]. In contrast, combined RAF/MEK inhibition can lead to more efficient ERK pathway inhibition/downregulation in all types of BRAF mutant cells [6, 8, 9, 11, 19, 45].

Overall, our results indicate that combined type II pan-RAF inhibition and MEK inhibition in BRAF mutant cells is efficient irrespective of BRAF mutation type. Type II pan-RAF inhibition induces no or weaker paradoxical ERK activation and predicts better efficacy with respect to early adaptive insensitivity. Type II pan-RAF inhibition is also more efficient against adaptive and innate RAF homodimer signaling and innate CRAF-dependent signaling. Our observations in non-V600 BRAF mutant cells are consistent with those of Whittaker, *et al.* [19] and Yao, *et al.* [17], who report high efficacies for type II RAF inhibitors against high-kinase BRAF mutants.

The present study supports the exploration of type II RAF inhibitors for treatment of tumors harboring BRAF mutations, including the previously poorly investigated impaired-kinase mutations. This class of driver oncogenic BRAF mutations [9, 14, 17] accounts for approximately half of the BRAF mutations in lung cancer [9, 17, 26]. A new generation of type II RAF inhibitors, such as LY3009120, is emerging [17, 28] that offer more efficient and safer RAF targeting compared to currently available type I RAF-inhibitors assessed in BRAF and KRAS mutant preclinical models [28, 46]. While LY3009120 as a single agent has not delivered satisfactory clinical results in BRAF and KRAS mutant tumors, probably due to poor pharmacodynamic responses [47], other type II inhibitors are in development [48]. Years of RAF targeting in V600E BRAF cancers has revealed that dual RAF/MEK targeting can produce better clinical outcomes [8, 49–51]. Our study justifies the further exploration of type II pan-RAF inhibitors in combination with Trametinib against lung (and probably other) cancers harboring different types of BRAF mutations.

## MATERIALS AND METHODS

### Cell lines and inhibitors

HEK293T cells were kindly provided by Prof. Ron Kooijman (FARC, Vrije Universiteit Brussel) and were cultured in Dulbecco’s Modified Eagles Medium (DMEM) (Life Technologies, 31966-047) supplemented with 10% fetal bovine serum (FBS) (Perbio Science, SV30160.03) and 100 U/ml penicillin 100 μg/ml streptomycin (pen-strep) (Life Technologies, 15140-148). H1666 cells were purchased from the ATCC (CRL-5885) and cultured in F12-based (ATCC: 30-2006) ACL-4 medium supplemented with 10% FBS and pen-strep. Both cell lines were tested periodically for mycoplasma infection, and all tests were negative. Dabrafenib (Tafinlar) was provided by GlaxoSmithKline (UK). Trametinib (S2673) and AZ628 (S2746) were obtained from Selleckchem (USA).

### Transfection and DNA plasmids

HEK293T cells (50,000–150,000 cells/well) were seeded in 24-well plates in antibiotic-free medium 24 h prior to transfection. Transfections and co-transfections were performed using Lipofectamine-2000 (116680-19) according to the manufacturer’s instructions. Medium was changed to OptiMEM and incubated for 30 min at 37° C before transfection. For experimental purposes, increasing CRAF expression while maintaining BRAF expression was achieved by co-transfecting 0.2 or 0.6 μg CRAF plasmids together with 0.2 μg BRAF plasmids. In other transfection experiments, 0.4 μg of each plasmid was transfected. After 6 h incubation, OptiMEM was changed to antibiotic-free DMEM (supplemented with 10% FBS). Cells were lysed and collected 48 h post-transfection for Western blot analysis. Transfection experiments were performed twice independently.

Recombinant BRAF expression cassettes were generated as previously described [6]. Briefly, a full-length V600E BRAF cDNA-bearing cassette (a gift from Loredana Vecchione of the Catholic University Leuven) was PCR-cloned (AccuPrime, Life Technologies, 12344-024) into the destination vector, PX3FLAG-CMV-14 (Sigma, E7908). WT and mutant BRAF plasmids were generated through site-directed mutagenesis (GeneArt Site-Directed Mutagenesis System, Life-Technologies, A13312). The full-length BRAF coding region and the insertion sites within expression vectors were sequenced. Empty vector (puno1) and HA-tagged CRAF expression vector (puno1-HA-hRAF1) were purchased from InvivoGen (Toulouse, France).

### Western blotting

At the indicated time-points, cells were washed with PBS and lysed in 1% triton-X buffer supplemented with 1% phosphatase inhibitor cocktail 2 (Sigma, P5726), 1% protease inhibitor cocktail (Sigma, P8340), and leupeptin trifluoroacetate (Sigma, L2023). Protein concentrations were determined using the Bradford protein assay kit (Bio-Rad), and equal amounts of protein were loaded on 10% resolving polyacrylamide gels (Mini-PROTEAN Tetra Cell). Proteins were transferred to polyvinylidene fluoride (PVDF) membranes overnight at 4° C. Membranes were blocked with Tris-Buffered Saline and Tween 20 (TBST) containing 5% non-fat milk. Blocked membranes were labelled with primary antibody overnight at 4° C, followed by 1 h incubation with the corresponding secondary horseradish peroxidase (HRP)-conjugated antibody at 37° C. HRP signal was detected using enhanced chemiluminescence (ECL) detection reagent (Isogen Life Science, K-12045-D20) and exposed on Fuji super films (104253). HA-CRAF and FLAG-BRAF were detected by Odyssey^®^ Fc Imaging System (LI-COR). Western blot antibodies were: phospho(p)-MEK1/2 (Cell Signalling, 9121), total MEK1/2 (Cell Signalling, 9122), p-ERK1/2 (Cell Signalling, 4370), total ERK1/2 (Cell Signalling, 4695), HA-TAG (Cell Signaling, 2367), FLAG (Sigma, F1804), and β-actin (Sigma, A1978).

### Cell viability and caspase 3/7 activity assays

Cells were seeded in white 384-well plates at 1000 cells/well for both viability and caspase 3/7 assays. Drugs were added at the indicated concentrations 24 h post seeding. After 72 h of drug treatment, viability and caspase 3/7 were determined using the CellTiter-Glo Luminescent Kit (Promega: G7570) and Caspase-Glo 3/7 Assay Kit (Promega; G8091), respectively, according to the manufacturer’s instructions. Caspase signals were normalized to the amount of viable cells in corresponding conditions in the same experiment.

### Confluency measurements

H1666 cells were seeded in 96-well plates at low density (1000 cells/well) and cultured overnight. One day later, inhibitors were added and cells were monitored for seven days using the Incucyte Zoom system (Essen Bioscience). Four images per well were taken at 1 h intervals. Confluency analyses were conducted using Incucyte Zoom software (Essen Bioscience) and normalized to DMSO-treated controls.

### Statistical methods

Viability and caspase 3/7 data corresponding to 72-h drug treatments show the means of two independent experiments, each performed in six replicates. Significance was determined using one-way ANOVAs with Tukey’s post-hoc analysis. We compared the viabilities and confluencies of two combinatorial drug treatments using the *t*-test. Viability and Incucyte data corresponding to 7-d drug treatments show the means of four independent experiments, each performed in four replicates.

## SUPPLEMENTARY MATERIALS FIGURE


