# Supplementary material for: Type II RAF inhibitor causes superior ERK pathway suppression compared to type I RAF inhibitor in cells expressing different BRAF mutant types recurrently found in lung cancer
Source: Oncotarget. 2018 Feb 27;9(22):16110–23. doi: 10.18632/oncotarget.24576 (PMC5882321; doi:10.18632/oncotarget.24576)
Supplement: Supplementary file 1 [file oncotarget-09-16110-s001.pdf]

Type II RAF inhibitor causes superior ERK pathway suppression compared to type I RAF inhibitor in cells expressing different BRAF mutant types recurrently found in lung cancer

SUPPLEMENTARY MATERIALS

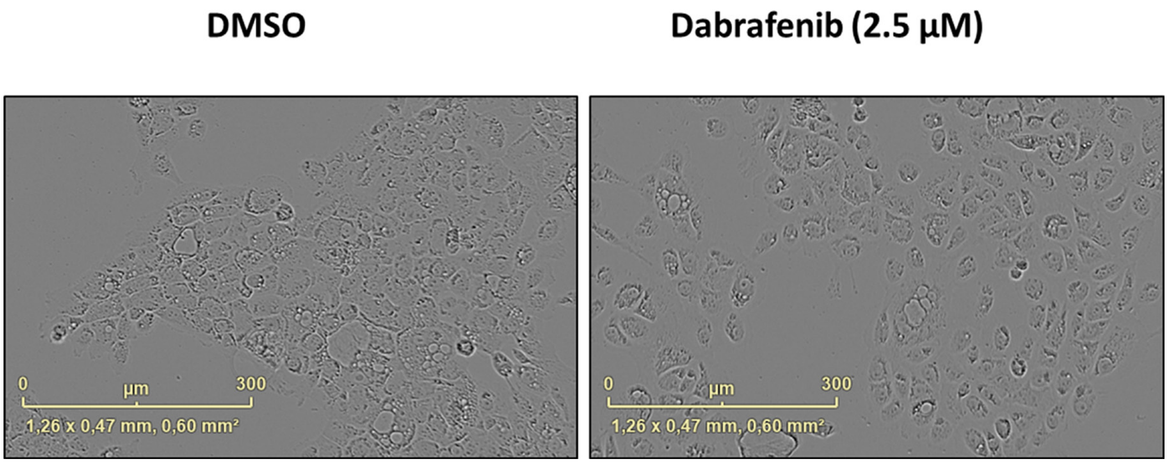

Supplementary Figure 1: High magnification images of H1666 cells treated with DMSO or Dabrafenib. High magnification images of Figure 6C reveal that H1666 cells treated with Dabrafenib for one week are less densely distributed compared to the DMSO group.
